# Targeting VPS4 elicits STING-driven anti-tumor immunity to suppress rhabdomyosarcoma growth

**DOI:** 10.1038/s41388-026-03800-1

**Published:** 2026-04-24

**Authors:** Ray Zhang, Longgui Chen, Xinwen Liang, Jiawen Zhang, Kouta Hamamoto, Tatsuya Hattori, Venugopal Vangala, Todd D. Schell, Giselle Saulnier Sholler, Yoshinori Takahashi, Hong-Gang Wang

**Affiliations:** 1 Division of Pediatric Hematology and Oncology, Department of Pediatrics, The Pennsylvania State University College of Medicine, Hershey, PA USA; 2 Department of Cell and Biological Systems, The Pennsylvania State University College of Medicine, Hershey, PA USA

**Keywords:** Tumour immunology, Cell signalling

## Abstract

The AAA+ ATPase VPS4 drives the ESCRT machinery in diverse intracellular membrane remodeling events, including endocytic receptor sorting, membrane repair, and autophagosome closure. Tumor cells often lose one VPS4 paralog (VPS4A or VPS4B), making them dependent on the remaining enzyme and creating a potential therapeutic vulnerability. Inhibiting VPS4 induces cancer cell-autonomous death and may also modulate the immune microenvironment, although the underlying mechanisms remain unclear. Here, we report that VPS4 inhibition triggered upregulation of cytokine and innate immune signaling, along with canonical NF-κB, stress response, and cell death pathways in murine rhabdomyosarcoma (RMS) cells. Pharmacological and genetic analyses identified the cGAS-STING-TBK1-IRF3 axis, activated by cytoplasmic mitochondrial DNA, as the primary driver of cytokine induction. In an orthotopic syngeneic RMS model, VPS4 inhibition suppressed tumor growth while fostering a more immunogenic microenvironment. Although STING was dispensable for VPS4 inhibition-induced RMS cell death, its loss reduced natural killer and dendritic cell infiltration and attenuated the overall anti-tumor effects of VPS4 inhibition. These findings establish a dual role for VPS4 inhibition in inducing tumor cell death and promoting anti-tumor immunity, highlighting the therapeutic potential of targeting VPS4 vulnerability in cancer.

## Introduction

The endosomal sorting complex required for transport (ESCRT) is a conserved membrane scission machinery essential for remodeling membranes in numerous cellular processes, including multivesicular endosome biogenesis, autophagosome closure, cytokinetic abscission, viral budding, nuclear envelope reformation, and plasma membrane and lysosome repair [1]. ESCRT comprises four core subcomplexes: ESCRT-I, ESCRT-II, ESCRT-III, and vacuolar protein sorting-associated protein 4 (VPS4). ESCRT-I and ESCRT-II coordinate the recruitment of ESCRT-III, which assembles into filaments at membrane constriction sites. VPS4 then remodels and disassembles ESCRT-III filaments to induce membrane scission and recycle ESCRT-III components. Disruption of ESCRT triggers activation of various pro-death pathways, leading to induction of apoptosis, necroptosis, and pyroptosis [2–7].

Beyond its roles in membrane remodeling and cell death regulation, recent studies have highlighted the roles of ESCRT machinery in immune signaling. Inhibition of ESCRT-mediated degradation of cytokine receptors leads to ligand-independent, constitutive activation of inflammatory NF-κB signaling [8]. ESCRT inhibition–induced cell death also promotes the release of immunomodulatory molecules and damage-associated molecular patterns [6, 9]. Loss of the ESCRT-III subunit charged multivesicular body protein 2 A (CHMP2A) enhances natural killer (NK) cell-mediated cytotoxicity by suppressing the secretion of pro-apoptotic extracellular vesicles [10]. More recently, ESCRT has been shown to negatively regulate stimulator of interferon genes (STING) signaling by sequestering STING-containing vesicles within endolysosomes [11, 12]. STING signaling, initiated by cyclic GMP-AMP synthase (cGAS) upon sensing cytosolic DNA, activates TANK-binding kinase 1 (TBK1) to upregulate the expression of type I interferons (IFN-α/β) and other inflammatory cytokines (e.g., CXCL10) through the interferon regulatory factors 3 and 7 (IRF3/IRF7) and NF-κB transcription factors [13, 14]. Thus, targeting ESCRT in tumors may not only induce cancer cell death but also engage anti-tumor immune responses.

The AAA+ ATPase VPS4 is the sole known active enzyme within the ESCRT machinery. Mammalian cells express two paralogs, VPS4A and VPS4B, which share ~80% sequence identity [15] and function redundantly in multiple ESCRT-mediated pathways [6, 7]. Studies of cancer genomes have revealed that VPS4A or VPS4B frequently undergo loss of heterozygosity across multiple tumor types, rendering cancer cells dependent on the remaining paralog for survival [6, 7]. Consistently, although no specific VPS4 inhibitors are currently available, a derivative of DBeQ, an ATP-competitive inhibitor of VPS4 and the related AAA+ ATPase valosin-containing protein [16, 17], has been shown to preferentially induce cell death in VPS4A- or VPS4B-deficient cancer cells compared to VPS4-intact cells [18]. These studies highlight VPS4 as a promising vulnerability in cancer.

Rhabdomyosarcoma (RMS) is one of the most common pediatric solid tumors [19] and has been shown to exhibit high sensitivity to VPS4 synthetic lethality [7]. Here, to establish the therapeutic potential of targeting VPS4 in cancer, we employed an orthotopic immunocompetent mouse model with syngeneically transplantable RMS cell lines. This approach allowed us to investigate the effects of a doxycycline-inducible VPS4 inhibition (mimicking a therapeutic intervention) on RMS growth within a functional immune system in vivo. Our findings show that VPS4 inhibition intrinsically upregulated cytokine expression through the cGAS–STING–TBK1–IRF3 axis and suppressed RMS growth. Notably, while VPS4 inhibition-induced cell death occurred independently of STING expression, the absence of STING1 in RMS tumors significantly attenuated the anti-tumor response elicited by VPS4 inhibition. Collectively, these findings reveal a dual role of VPS4 inhibition in promoting cancer cell death and activating anti-tumor immunity, underscoring the therapeutic potential of targeting VPS4 in cancer.

## Materials and Methods

### Reagents

The following antibodies were used for immunoblotting: GFP (Cell Signaling Technology [Danvers, MA, USA], #2956S, 1:1000), IRF3 (Cell Signaling Technology #4302S, 1:1000), phospho-TBK1 (Cell Signaling Technology #5483S, 1:1000), TBK1 (Cell Signaling Technology #71543SF, 1:1000), phospho-STING (Cell Signaling Technology #72971S, 1:1000), STING (Cell Signaling Technology #13647S, 1:1000), Cleaved caspase 3 (Cell Signaling Technology #9661S, 1:1000), β-actin (Sigma-Aldrich [St. Louis, MI, USA], #A5441, 1:10,000), VPS4A (Sigma-Aldrich #SAB4200215, 1:1000), VPS4B (Santa Cruz Biotechnology [Dallas, TX, USA], #sc-377162, 1:200), cGAS (Cell Signaling Technology #31659S, 1:1000), p62/SQSTM1 (PROGEN Biotechnik [Heidelberg, Germany], #GP62-C, 1:1000), ATF-3 (Cell Signaling Technology #18665S, 1:1000), and LC3B (Novus Biologicals [Centennial, CO, USA] #NB100-2220, 1:5000).

The following inhibitors were used to dissect innate immune signaling pathways: BMS-345541 (Sigma-Aldrich #B9935-5MG), 5Z-7-oxozeaenol (MedChemExpress [Monmouth Junction, NJ, USA], #HY-12686), GSK8612 (MedChemExpress #HY-111941), C-176 (MedChemExpress #HY-112906), NBP2-31226 (Novus Biologicals #NBP2-31226), RIG012 (MedChemExpress #HY-147124), and MSA-2 (MedChemExpress #HY-136927).

### RNA-seq and gene enrichment analysis

Total RNA samples were extracted using RNeasy Plus Mini Kit (Qiagen [Germantown, MD, USA], #74136) and mRNA-seq was performed by Novogene Corporation, Inc (Beijing, China). In brief, cDNA libraries were prepared with NEBNext Ultra II Directional RNA Library Prep Kit for Illumina (E7760, New England Biolabs [Ipswich, MA, USA]) and evaluated using Qubit and real-time PCR for quantification, and an Agilent Bioanalyzer for size distribution. Quantified libraries were pooled and sequenced on an Illumina NovaSeq 6000 platform using PE150 reads, based on effective library concentration and target data output. Raw FASTQ reads were processed with FASTQ software to remove adapter sequences, reads containing poly-N, and low-quality reads. The trimmed FASTQ files were aligned to the Mus musculus reference genome (mm10) using HISAT2 (v2.0.5), and gene-level read counts were obtained with featureCounts (v1.5.0-p3). The limma-voom method implemented on the iDEP platform [20] was used to normalize gene count data and identify differentially expressed genes. Gene counts and limma-voom–normalized expression values are provided in Tables S1 and S2, respectively. Gene set enrichment analysis (GSEA, v4.4.0) and Cytoscape (v3.10.4) were used for pathway enrichment and enrichment map generation, respectively. Heatmaps of differentially expressed genes were generated using Heatmapper (www.heatmapper.ca).

### Cell culture, transfection, and viral transduction

Mouse RMS cell lines KMR19 and KMR46, provided by Dr. Jason Yustein (Baylor College of Medicine), were derived from tumors in a genetically engineered mouse model generated by crossing MYOD1-Cre mice with germline floxed p53, Lox-Stop-Lox (LSL)-p53^R172H^, or LSL-K-Ras^G12D^ alleles [21]. Both lines were established on a C57BL/6J background, and harbor the K-Ras^G12D^ mutation. KMR46 carries a p53^-/+^ genotype, while KMR19 carries p53^R172H/+^. Cells were checked for mycoplasma using the MycoStrip® - Mycoplasma Detection Kit (InvivoGen). Cells were cultured in RPMI 1640 medium (Corning #10-040-CV) supplemented with 10% v/v FBS (Thermo Fisher Scientific [Waltham, MA, USA], #A4736101) and 100 U/mL penicillin and streptomycin (Thermo Fisher Scientific #SV3007901) [21]. For mitochondrial DNA depletion, the medium was further supplemented with 1 µg/mL ethidium bromide (EtBr; Invitrogen #15585011) [22]. VPS4B-KO, STING1-KO, and IRF3-KO KMR46 cells were generated by CRISPR-Cas9-mediated gene editing. Additional details are available in the Supplemental Materials.

### Construction of RMS cells with inducible DN-VPS4A expression

DN-VPS4A-expressing KMR19 and KMR46 cells were generated by transducing each line with lentiviral particles harboring doxycycline-inducible dominant-negative (DN) VPS4A (VPS4A^E228Q^; pCDH-TRE3G-GFP-DNVPS4A-EF1-PuroR-P2A-T2A-Tet-on-3G) followed by puromycin selection (10 µg/mL). GFP- or GFP-tagged wild-type VPS4A (WT-VPS4A)-expressing cells were generated similarly using lentiviral particles harboring GFP (pCDH-TRE3G-GFP-PuroR-P2A-T2A-Tet-on-3G) or WT-VPS4A (pCDH-TRE3G-GFP-VPS4A-EF1-PuroR-P2A-T2A-Tet-on-3G) followed by puromycin selection. All expression plasmids were assembled into the pCDH1 vector (System Biosciences [Palo Alto, CA, USA]) using Gibson Assembly (New England Biolabs #E2621). Target gene expression was driven by the TRE3G promoter, with the Tet-On 3G transactivator co-expressed via tandem P2A-T2A linkers and the EF1α-puromycin cassette. All constructs were confirmed by sequencing.

### Immunoblotting

Cells were lysed in radio-immunoprecipitation buffer (25 mM Tris-HCl pH 8.0, 150 mM NaCl, 1 mM EDTA, 1% v/v IGEPAL CA-630, 0.5% w/v sodium deoxycholate, and 1% v/v SDS) containing protease (1:250) and phosphatase inhibitor cocktails (1:100). Lysates were resolved via SDS-PAGE and probed with the indicated antibodies. Signals were acquired with the Odyssey CLx (LI-COR [Lincoln, NE, USA]) for fluorescence or Odyssey XL (LI-COR) for chemiluminescence detection and visualized in Image Studio version 6 (LI-COR).

### Cell death assays

For live-cell quantification, cells seeded on clear-bottom 96-well plates were incubated with or without 1 µg/mL doxycycline (Sigma-Aldrich #D9891) in the presence of Incucyte® Cytotox NIR Dye (Sartorius [Bohemia, NY, USA], #4846, 1:2000). Fluorescent images were obtained every 2 h using the Incucyte SX5 Live-Cell Analysis System (Sartorius, 10X objective) and analyzed using the Cell-by-Cell Analysis Adherent module of the IncuCyte SX5 Software (Sartorius, Version 2022B Rev2). Cell death was quantified as the percentage of GFP- and NIR-double-positive cells relative to the total GFP-positive population.

### RT-qPCR

Total RNA was extracted using the RNeasy Plus Mini Kit (Qiagen #74136) according to the manufacturer’s instructions. cDNA was synthesized using the QuantiTect Reverse Transcription Kit (Qiagen #205313). Target cytokines were amplified using a standard qPCR protocol: cDNA was diluted 1:20 and mixed with iTaq Universal SYBR Green Supermix (Bio-Rad Laboratories [Hercules, CA, USA], #1725121), 10 µM forward and reverse primers, and sterile distilled water. Primers for mouse PPIB, CXCL10, and IFNB1 were purchased from Integrated DNA Technologies (Coralville, IA, USA) as PrimeTime Predesigned qPCR Assays: PPIB (Mm.PT.58.29807961), CXCL10 (Mm.PT.58.43575827), and IFNB1 (Mm.PT.58.30132453.g). Amplification and data collection were performed on a Bio-Rad CFX96 Real-Time System using the following program: 95 °C for 2 min (initial denaturation), followed by 40 cycles of 95 °C for 5 s and 60 °C for 30 s, and a final melt curve step. All samples were run in triplicate, and assays were repeated at least three times. Quantification cycle (Cq) values for the cytokines were normalized to the PPIB housekeeping gene, and data were expressed as fold change over vehicle-control sample.

### Tumor engraftment and monitoring

KMR46 cells (5 × 10³ per mouse) were intramuscularly injected into the left gastrocnemius muscle of 6–8-week-old female C57BL/6J mice (RRID:IMSR_JAX:000664). Mice were fed 625 ppm doxycycline-infused chow (INOTIV [Lafayette, IN, USA], #TD.120769) starting either 1 day after injection (Fig. 7) or once tumors reached 300–500 mm^3^ (Fig. 8). In the experiment shown in Fig. 7, all mice (*n* = 15 per group) received doxycycline-infused chow continuously for 3 weeks or until control tumors reached ~3000 mm³. In the experiment shown in Fig. 8, mice from each cell-injected group were randomized to receive either standard or doxycycline-infused chow (*n* = 6 per condition) for 1 week or until control tumors reached ~1500–2000 mm³. Tumor growth was measured every 3-4 days using calipers, and tumor volume (mm^3^) was calculated by formula: 0.5 x length x width^2^. Mice were housed and handled in accordance with the Guide for the Care and Use of Laboratory Animals. All procedures were performed under a protocol (IACUC #PRAMS201145989) approved by the Institutional Animal Care and Use Committee at the Pennsylvania State University College of Medicine.

### Tumor immune profiling by flow cytometry

Tumors were harvested at the experimental endpoint, minced, and digested in a collagenase-DNAse mixture (1 mg/mL collagenase [Gibco [Billings, MT, USA], #17018-029] + 400 µg/mL DNase I [Sigma-Aldrich #9003-98-9] in PBS supplemented with 10% FBS) at RT for 1 h. The digested tissues were passed through 70 µm mesh screens using a 5 mL-syringe plunger to prepare single-cell suspensions. Cells were centrifuged (300 g, 5 min), resuspended in 1X RBC lysis buffer (eBioscience [San Diego, CA, USA], #00-4300-54), and incubated at RT for 10 min. After a second centrifugation (300 g, 5 min), cell pellets were resuspended in FACS buffer (PBS supplemented with 2% FBS) and filtered twice through 5 mL polystyrene round-bottom tubes with cell-strainer caps. For staining, cells were washed twice with DPBS, blocked with anti-mouse CD16/CD32 Fc block (BioLegend [San Diego, CA, USA], #101302), and incubated with the live/dead fixable viability dye Alexa Fluor 780 (BD Biosciences #565388) at RT for 15 min in the dark. Following a wash with FACS buffer, cells were stained with the antibody panels listed in the Supplementary Materials at RT for 15 min in the dark (at 1:100 dilution). The gating strategy was described previously [23]. Data were acquired on the Fortessa instrument at Penn State College of Medicine’s Flow Cytometry Core and analyzed using FlowJo v.10.8.1 software.

### Statistical analysis

GraphPad Prism (GraphPad Software) was used for statistical analyses. Two-tailed unpaired Student’s *t*-tests were applied for single comparisons. One-way or two-way analysis of variance (ANOVA) followed by Tukey’s, Sidak’s, or Fisher’s LSD post hoc tests were conducted for multiple comparisons. All data from in vitro studies are representative of at least three independent experiments. For animal studies, the sample size was determined using a two-sample t-test analysis at the test power of 0.80, set significance level α = 0.05, and an effect size based on preliminary experiments.

## Results

### VPS4 inhibition induces cell death accompanied by the upregulation of cytokine and immune signaling pathways in RMS cells

**Fig. 1:**
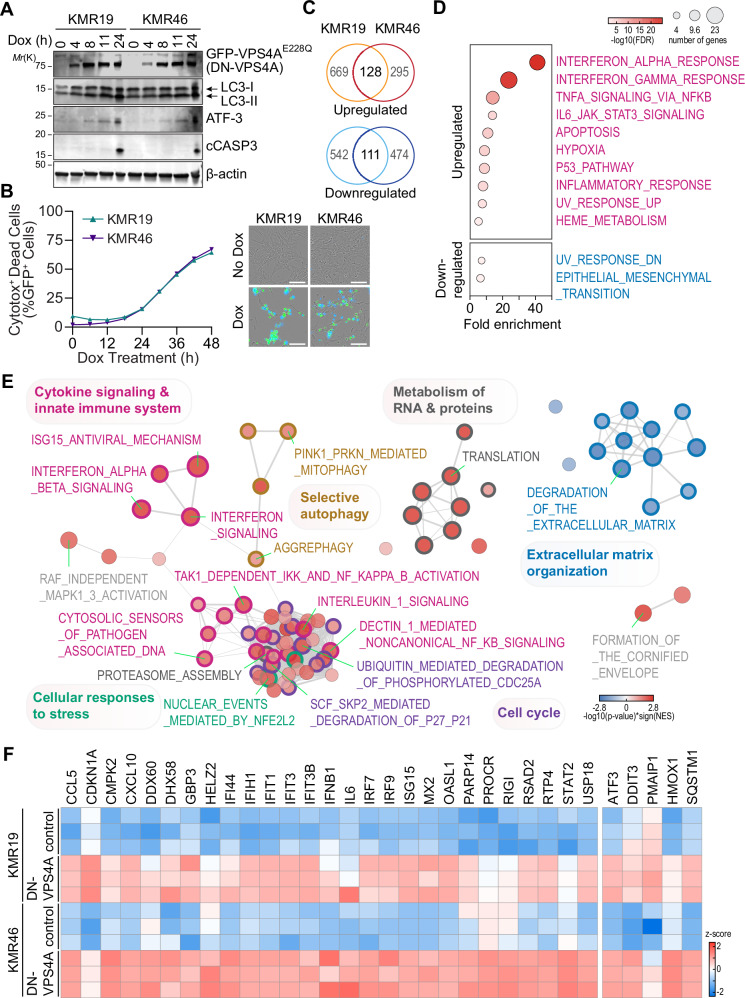
VPS4 inhibition triggers cell death and activates cytokine signaling, innate immune, and stress response pathways in RMS cells. **A** Immunoblot analysis of KMR19 and KMR46 cells transduced with a doxycycline (Dox)-inducible, GFP-tagged dominant-negative VPS4A (VPS4A^E228Q^; DN-VPS4A) following treatment with 1 µg/mL Dox for the indicated periods of time. **B** Time-lapse monitoring of DN-VPS4A-expressing (GFP^+^; green) cells undergoing cell death, marked by Cytotox positivity (blue), after Dox treatment (left). Data are presented as mean ± SEM. Representative images at 48 h are shown (right). Scale bar represents 100 µm. **C** Venn diagram of genes differentially upregulated (top) or downregulated (bottom) in DN-VPS4A-expressing cells compared with control GFP following 10 h of Dox treatment (fold change [FC] > 2, false discovery rate [FDR] < 0.1). **D** Dot plot of Hallmark pathways enriched for genes commonly upregulated or downregulated identified in (**C**). **E** Enrichment map of Reactome pathways identified by GSEA in cells treated with Dox for 10 h (DN-VPS4A vs control GFP; FDR < 0.25). Nodes represent individual pathways, edges indicate gene set overlap, and node color represents the significance and direction of enrichment for each pathway (-log_10_(*p*-value) x sign[NES]). **F** Heatmap of genes upregulated by DN-VPS4A compared to control GFP. Genes on the left correspond to cytokine and innate immune response pathways, while those on the right include stress response and apoptosis genes previously linked to ESCRT inhibition.

To evaluate the impact of VPS4 inhibition on tumor progression, a doxycycline-inducible, GFP-tagged dominant-negative form of VPS4 (VPS4A^E228Q^; DN-VPS4A) [24] was transduced into two murine G12D mutant K-RAS-driven RMS cell lines: KMR19, which harbors a p53^R172H^ allelic mutation, and KMR46, which is a p53 heterozygous knockout. These cell lines were chosen for their representation of common genetic alterations in fusion-negative RMS and for their ability to undergo orthotopic and syngeneic transplantation, faithfully recapitulating the molecular and histopathology features of human disease [21]. Immunoblot analysis revealed DN-VPS4A expression as early as 4 h after 1 µg/mL doxycycline treatment, accompanied by accumulation of the autophagosomal membrane marker LC3-II and the stress-responsive transcription factor ATF3 in both RMS cell lines (Fig. 1A). Cleavage of CASP3 became evident by 24 h, indicating the induction of apoptosis. Time-lapse imaging further confirmed the onset of cell death after 24 h of doxycycline exposure (Fig. 1B). These findings validate the vital role of ESCRT in maintaining autophagic flux and restraining cellular stress and death signaling [3, 24, 25].

**Fig. 2:**
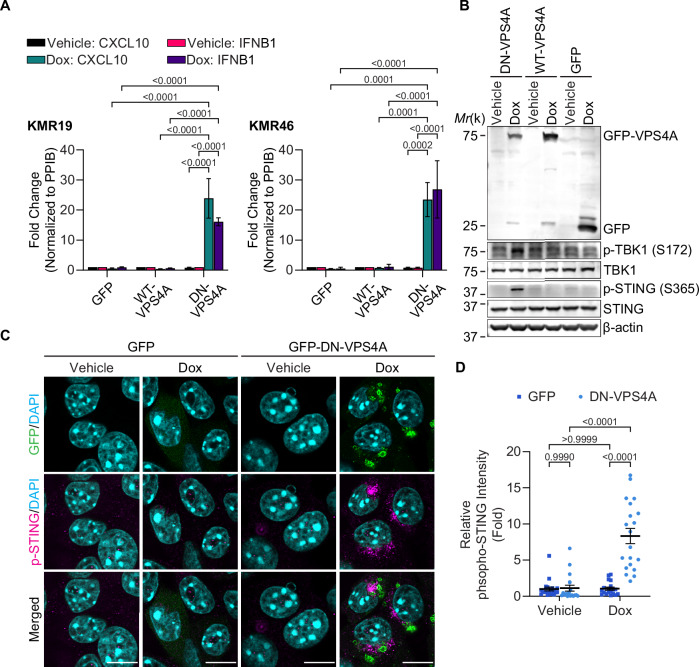
Inhibition of VPS4 activates the STING pathway in RMS cells*.* **A** RT-qPCR analysis of CXCL10 and IFNB1 expression in KMR19 (left) and KMR46 (right) cells transduced with doxycycline (Dox)-inducible, GFP-tagged wild-type (WT) or dominant-negative (DN) VPS4A, or GFP-only control, following treatment with 1 µg/mL Dox or vehicle (water) for 10 h (*n* = 3). **B** Immunoblot analysis of the indicated KMR46 cells treated with or without Dox for 10 h. **C** Representative confocal images of the indicated KMR46 cells treated with or without Dox for 10 h. Five random fields of view were taken per sample. Scale bar represents 10 µm. **D** Quantification of phospho-STING fluorescence from (**C**). Each data point represents an individual cell. Data in (**A**) and (**D**) are presented as mean ± SEM. Statistical significance was determined by two-way ANOVA followed by Tukey’s multiple-comparisons test.

To characterize the gene expression signature associated with VPS4 inhibition-induced RMS cell death, DN-VPS4A-transduced KMR19 and KMR46 cells were treated with doxycycline or vehicle (water) for 10 h before being subjected to RNA sequencing. Differential gene expression analysis identified 1450 (797 upregulated and 653 downregulated) genes altered by DN-VPS4A induction in KMR19 cells, and 1008 (423 upregulated and 585 downregulated) genes altered in KMR46 cells (Fig. 1C). Of these differentially expressed genes (DEGs), 128 and 111 genes were commonly upregulated and downregulated, respectively, in both RMS cell lines (Fig. 1C). Hallmark pathway analysis of the commonly upregulated gene set revealed strong enrichment in cytokine signaling and inflammatory response pathways, including interferon-α/γ response, TNF-α signaling via NF-κB, IL6-JAK-STAT3 signaling, followed by apoptosis. Conversely, the commonly downregulated gene set showed modest enrichment in UV response downregulation and epithelial-mesenchymal transition pathways (Fig. 1D). Gene Set Enrichment Analysis (GSEA) in KMR46 cells corroborated these findings, showing significant enrichment in cytokine signaling and innate immune system pathways upon VPS4 inhibition (Fig. 1E). Additional GSEA pathways positively enriched upon VPS4 inhibition included NFE2L2/NRF2-mediated cellular response (including upregulation of HMOX1 and SQSTM1), selective autophagy, RNA and protein metabolism, proteasome assembly, and cell cycle regulation, while extracellular matrix organization was negatively enriched (Fig. 1E, F). Upregulation of ATF3, DDIT3, and PMAIP1 was also detected, consistent with the induction of cellular stress and mitochondrial apoptotic signaling upon ESCRT inhibition [3]. These pathway genes were also upregulated in KMR19 cells upon DN-VPS4A expression, though the magnitude of change was less pronounced for some genes (Fig. 1F). Notably, upregulation of cytokines, including the Type I interferon IFNB1 and the interferon-stimulated gene CXCL10, was observed only upon induction of DN-VPS4A, but not wild-type VPS4 or control GFP (Fig. 2A). Consistently, increases in the innate immune pathway markers phospho-STING and phospho-TBK1 were detected only upon DN-VPS4A expression (Fig. 2B). Confocal microscopy revealed the accumulation of GFP-tagged DN-VPS4A on vacuole-like structures as well as small puncta, suggesting disruption of endosomal sorting and autophagy. In parallel, clustering of phospho-STING signals was observed (Fig. 2C, D), consistent with activation of the STING pathway.

**Fig. 3:**
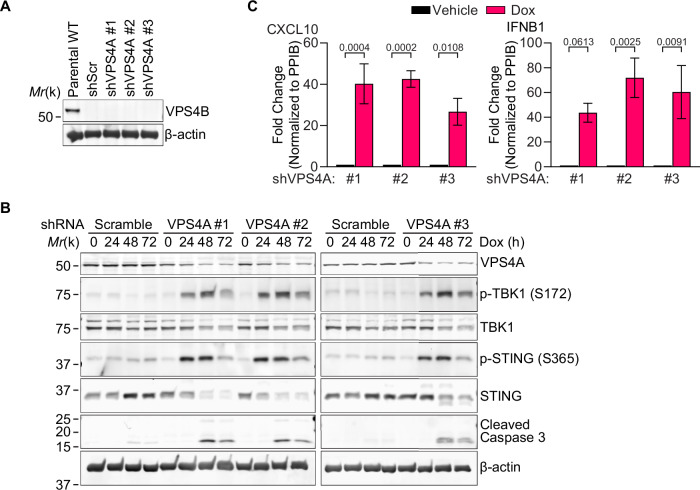
Depletion of VPS4 paralogs triggers innate immune signaling and apoptosis. **A**, **B** Immunoblot analysis of WT or VPS4B-knockout (KO) KMR46 cells transduced with Dox-inducible VPS4A shRNAs (shVPS4A #1-3) or a scrambled control shRNA (shScr). In (**B**), cells were treated with Dox for the indicated periods of time. **C** RT-qPCR analysis of CXCL10 (left) and IFNB1 (right) expression in KMR46 VPS4B-KO cells transduced with the indicated inducible shVPS4As and treated with Dox for 24 h (*n* = 3). Data in (**C**) is presented as mean ± SEM. Statistical significance was determined by two-way ANOVA followed by Sidak’s multiple-comparisons test.

The impact of VPS4 inhibition on immune response pathways was further evaluated by depleting both VPS4 paralogs, VPS4A and VPS4B. To this end, VPS4B-knockout (KO) KMR46 cells were generated using the CRISPR-Cas9 system and transduced with lentiviruses encoding doxycycline-inducible VPS4A shRNAs (shVPS4A#1-3) or a control scrambled shRNA (shScr) (Fig. 3A). Immunoblot analysis showed time-dependent reduction of VPS4A upon doxycycline treatment in cells transduced with any shVPS4A but not shScr. As expected, this VPS4A reduction was accompanied by increased expression of phospho-STING and phospho-TBK1 alongside cleavage of CASP3 (Fig. 3B). Consistently, upregulation of CXCL10 and IFNB1 was detected upon doxycycline treatment (Fig. 3C). Collectively, these results demonstrate that VPS4 inhibition activates cytokine and innate immune signaling concomitant with induction of RMS cell death.

### The STING-TBK1-IRF3 signaling axis mediates cytokine upregulation upon VPS4 inhibition

**Fig. 4:**
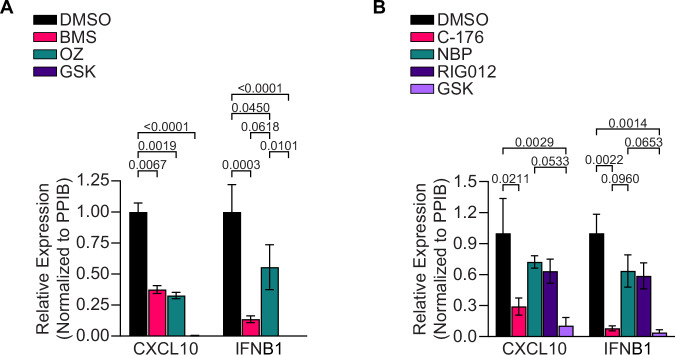
Pharmacological inhibition of TBK1 and STING markedly reduces CXCL10 and IFNB1 expression induced by VPS4 inhibition. RT-qPCR analysis of CXCL10 and IFNB1 expression in DN-VPS4A-transduced KMR19 cells pretreated for 1 h with BMS-345541 (BMS), 5Z-7-oxozeaenol (OZ), or GSK8612 (GSK) at 5 µM (**A**), or with C-176, NBP2-31226 (NBP), RIG012, or GSK at 1 µM (**B**), followed by addition of 1 µg/mL doxycycline for 10 h (*n* = 3). DMSO was used as the vehicle control for all pretreatments. Data are presented as mean ± SEM. Statistical significance was determined by two-way ANOVA followed by Tukey’s multiple-comparisons test.

Our data indicated that VPS4 inhibition induces activation of STING and TBK1 (Fig. 2A–D). Given that ESCRT has also been shown to limit NF-κB signaling by regulating cytokine receptor trafficking [8], we next sought to elucidate the mechanisms underlying cytokine upregulation upon VPS4 inhibition. To this end, we first examined the effects of pharmacological inhibition of TBK1 (GSK8612; GSK) [26], canonical NF-κB (BMS-345541; BMS) [27], and non-canonical NF-κB signaling (5Z-7-oxozeaenol; OZ) [28]. Treatment with GSK8612 almost completely blocked induction of CXCL10 and IFNB1 upon DN-VPS4A expression. Upregulation of both cytokines was also partially suppressed by BMS-345541 and 5Z-7-oxozeaenol, with BMS-345541 exerting a stronger inhibitory effect on IFNB1 (Fig. 4A). These results suggest that multiple signaling pathways contribute to cytokine upregulation upon VPS4 inhibition, with TBK1 serving as a primary driver.

TBK1 can be activated downstream of several innate immune pathways including STING, Toll-like receptors (TLRs), or RIG-I-like receptors (RLRs) [29, 30]. To identify the upstream pathway responsible for TBK1-dependent cytokine production upon VPS4 inhibition, we treated KMR19 cells with inhibitors targeting STING (C-176) [31], the RLR pathway (RIG012) [32], or the TLR pathway inhibitor (NBP2-31226; NBP; Novus Biologicals) [33]. We found that C-176 markedly reduced DN-VPS4A-induced CXCL10 and IFNB1 expression, whereas RIG012 and NBP2-31226 produced only modest attenuation (Fig. 4B). This suggests that STING, via STING-mediated activation of TBK1, is the dominant pathway driving cytokine upregulation upon VPS4 inhibition.

**Fig. 5:**
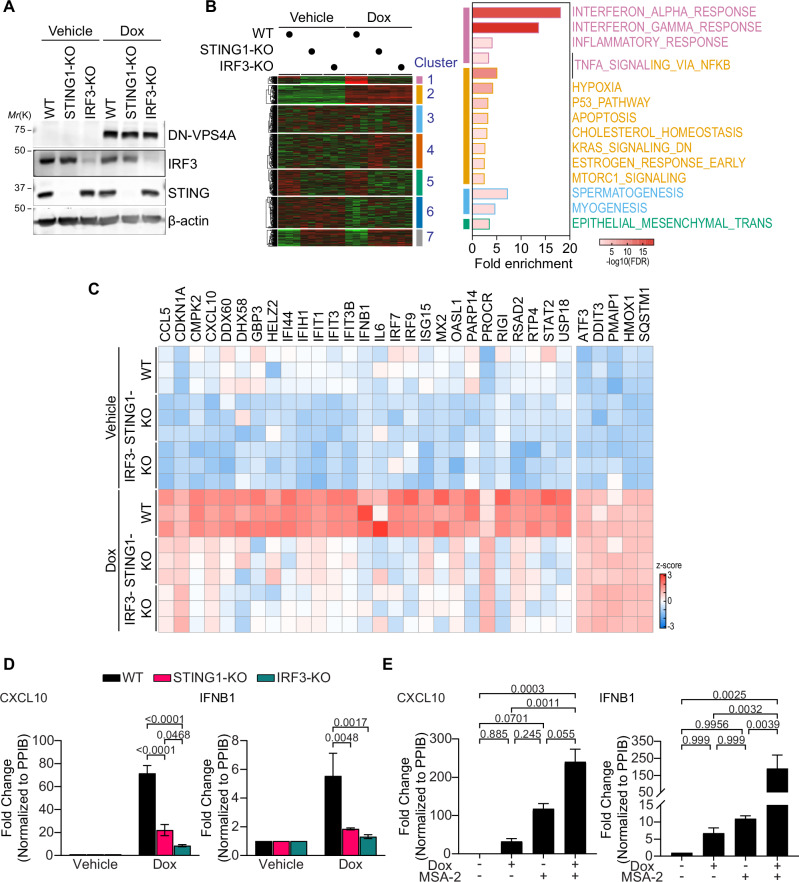
The STING-TBK1-IRF3 signaling axis is critical for inducing cytokine expression and innate immune signaling upon VPS4 inhibition. **A** Immunoblot analysis of WT, STING1-knockout (KO), and IRF3-KO KMR46 cells transduced with doxycycline (Dox)-inducible DN-VPS4A and treated with 1 µg/mL Dox for 10 h. **B** Heatmap and clustering of differentially expressed genes in DN-VPS4A-transduced KMR46 WT, STING1-KO, and IRF3-KO cells treated with Dox for 10 h (left; FDR < 0.1). Bar plot of Hallmark pathways enriched in identified clusters (right; FDR < 0.1). **C** Heatmap of genes upregulated by DN-VPS4A in cytokine and innate immune response pathways (left) alongside stress response and apoptosis genes previously linked to ESCRT inhibition (right). **D** RT-qPCR analysis of DN-VPS4A-induced CXCL10 and IFNB1 expression in KMR46 WT, STING1-KO, and IRF3-KO cells treated with Dox for 10 h (*n* = 3). **E** RT-qPCR analysis of DN-VPS4A-induced CXCL10 (left) and IFNB1 (right) expression in KMR46 cells treated with 1 µg/mL Dox alone for 10 h, 33 µM MSA-2 alone for 3 h, or pretreated with Dox for 7 h followed by MSA-2 for 3 h (*n* = 3). Data in (**D**) and (**E**) were presented as mean ± SEM. Statistical significance was determined by two-way ANOVA followed by Tukey’s (**E**) or Sidak’s (**D**) multiple-comparisons test.

To further validate the role of the STING pathway in VPS4 inhibition-induced cytokine production, we generated STING1-knockout (STING1-KO) and IRF3-knockout (IRF3-KO) DN-VPS4A-transduced KMR46 cells using CRISPR/Cas9 (Fig. 5A). Wild-type (WT), STING1-KO, and IRF3-KO DN-VPS4A-transduced KMR46 cells were treated with 10 h of doxycycline or vehicle (water) before being subjected to RNA sequencing. K-means clustering of the top 2000 DEGs revealed distinct gene clusters comprising both STING1- and IRF3-dependent and independent groups (Fig. 5B). Clusters 1 and 2 contained genes robustly upregulated by DN-VPS4A, with Cluster 1 being STING1- and IRF3-dependent, and Cluster 2 STING1- and IRF3-independent. Pathway analysis revealed that cytokine and inflammatory signaling pathways (IFN-α response; IFN-γ response; inflammatory response) were uniquely enriched in Cluster 1, while Cluster 2 was enriched for cellular stress (hypoxia) and cell death (apoptosis) pathways. NF-κB signaling (TNF-α signaling via NF-κB) appeared in both Clusters 1 and 2. Other clusters, such as Cluster 5 (STING1/IRF3-dependent, enriched for spermatogenesis and myogenesis) and Cluster 3 (STING1/IRF3-independent, enriched for spermatogenesis and myogenesis), were largely unaffected by DN-VPS4A expression. Consistent with these results, heatmap analysis of DN-VPS4A-induced genes indicated that most cytokine and immune signaling genes were STING1- and IRF3-dependent, although a subset of these genes, along with stress response and apoptosis genes, was upregulated even in the absence of STING1 or IRF3 (Fig. 5C). qPCR analysis further confirmed the critical role of the STING-IRF3 axis in DN-VPS4A-induced CXCL10 and IFNB1 expression, although a minor induction of CXCL10 persisted in STING1-KO and IRF3-KO cells (Fig. 5D). Notably, cytokine upregulation triggered by VPS4 inhibition was further enhanced by co-treatment with the STING agonist MSA-2 [34] (Fig. 5E), consistent with prior reports implicating ESCRT in terminating STING signaling [11, 12]. Collectively, these results indicate that while multiple signaling pathways are engaged upon VPS4 inhibition, the STING-TBK1-IRF3 axis serves as the dominant signaling mechanism driving cytokine production.

### Cytoplasmic mitochondrial DNA drives cytokine upregulation upon VPS4 inhibition

**Fig. 6:**
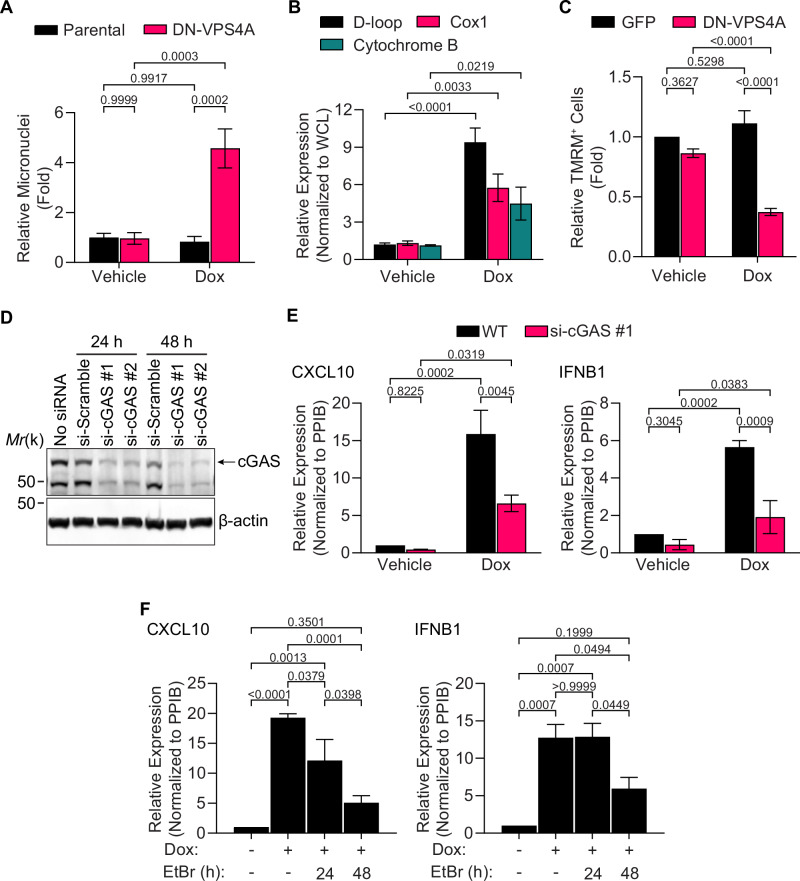
Cytoplasmic mtDNA is a key driver of VPS4 inhibition-induced CXCL10 and IFNB1 expression. **A** Flow cytometry quantification of micronuclei formation in KMR46 cells transduced with doxycycline (Dox)-inducible DN-VPS4A and treated with 1 µg/mL Dox for 24 h (*n* = 3). **B** qPCR quantification of cytosolic D-loop, COX1, and cytochrome B in DN-VPS4A-transduced KMR46 cells treated vehicle or 1 µg/mL Dox for 24 h (*n* = 5). **C** Flow cytometry quantification of TMRM-positive KMR46 cells transduced with Dox-inducible GFP or DN-VPS4A treated with vehicle or 1 µg/mL Dox for 10 h (*n* = 6). **D** Immunoblot analysis of DN-VPS4A-transduced KMR46 cells transfected with cGAS siRNAs (si-cGAS #1, #2) or a scrambled control siRNA (si-Scramble) for the indicated periods of time. **E** RT-qPCR analysis of CXCL10 (left) and IFNB1 (right) expression in DN-VPS4A-transduced KMR46 cells transfected with si-cGAS #1 for 48 h followed by Dox treatment for 10 h (*n* = 3). F RT-qPCR analysis of CXCL10 (left) and IFNB1 (right) expression in DN-VPS4A-transduced KMR46 cells pretreated with 1 µg/mL ethidium bromide (EtBr) for the indicated periods of time before replacing the media for 10 h of Dox treatment (*n* = 3). Data in (**A–C**), (**E**), (**F**) are presented as mean ± SEM. Statistical significance was determined by one-way ANOVA followed by Tukey’s multiple-comparisons test (**A**), two-way ANOVA followed by Sidak’s multiple-comparisons test (**B**), two-way ANOVA followed by Tukey’s multiple-comparisons test (**C**, **F**), or two-way ANOVA followed by Fisher’s LSD multiple-comparisons test (**E**).

Cytosolic DNA, derived from micronuclei generated by genomic instability or mitochondrial DNA (mtDNA) released by damaged mitochondria, is a potent activator of STING signaling [13, 14]. Given the critical roles of VPS4 in maintaining both nuclear envelope integrity [7, 35] and mitochondrial homeostasis [36], we examined whether cytosolic DNA contributes to cytokine upregulation upon VPS4 inhibition. As expected from prior studies [7, 35], DN-VPS4A expression led to significant accumulation of micronuclei in KMR46 cells (Fig. 6A). In parallel, a marked increase in cytoplasmic mtDNA, measured across multiple mtDNA regions, including the D-loop, COX1 and Cytochrome B, was detected following DN-VPS4A induction (Fig. 6B), and this increase was accompanied by a reduction in mitochondrial membrane potential, as indicated by decreased tetramethylrhodamine (TMRM) signals (Fig. 6C). To further verify the contribution of cytoplasmic DNA, we depleted cyclic GMP-AMP synthase (cGAS), the principal cytosolic DNA sensor that initiates STING signaling [13, 14]. As expected, siRNA-mediated depletion of cGAS attenuated the upregulation of both cytokines upon DN-VPS4A expression (Fig. 6D, E).

Because mtDNA is reportedly a more potent activator of STING signaling than nuclear DNA [37, 38], we next evaluated the contribution of cytoplasmic mtDNA using ethidium bromide (EtBr), which at nonlethal doses preferentially inhibits the replication and transcription of extrachromosomal DNA and is widely used to deplete mtDNA [22, 39, 40]. Pretreatment with EtBr for 24 h significantly reduced DN-VPS4A–induced CXCL10 expression, and this effect was more pronounced after 48 h of pretreatment (Fig. 6F). Consistently, the reduction of IFNB1 expression upon DN-VPS4A induction was also observed following 48 h of EtBr pretreatment. Together, these results indicate that cytoplasmic mtDNA, serves as a key driver of cytokine production following VPS4 inhibition.

### VPS4 inhibition impedes tumor growth and promotes an immunogenic tumor microenvironment

**Fig. 7:**
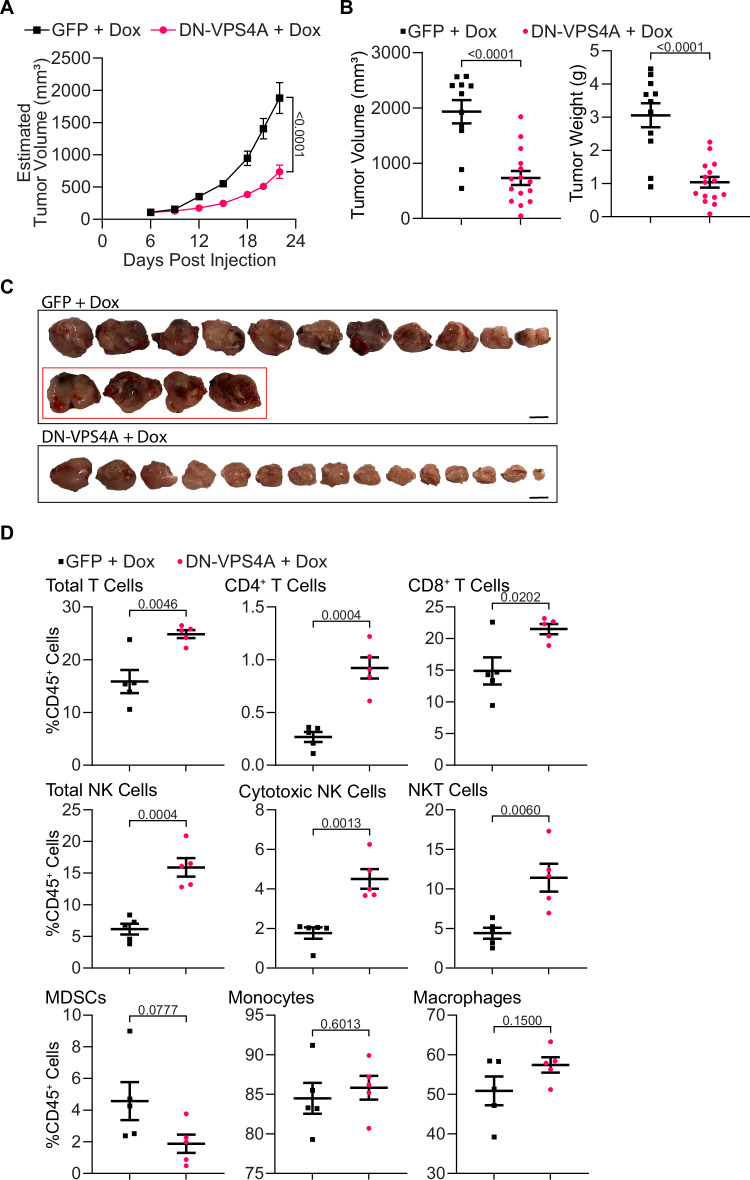
VPS4 inhibition reduces KMR46 tumor progression and enhances tumor infiltration by NK and T cell subsets. **A** Tumor growth curves of doxycycline (Dox)-inducible DN-VPS4A- or GFP-control-transduced KMR46 syngeneic allografts in immunocompetent C57BL/6 mice (*n* = 15 per group). Dox-infused feed (625 ppm) was administered to all mice beginning on day 1 post injection. **B** Tumor volumes and weights at the experimental endpoint (21 days post injection). **C** Representative images of harvested tumors. Scale bar represents 1 cm. Tumors bracketed in red were excluded from analysis in (**B**) and (**D**) due to being harvested one day prior to the rest. **D** Flow cytometry quantification of tumor-infiltrating lymphoid and myeloid subsets in representative DN-VPS4A- and GFP-control-transduced KMR46 tumors (*n* = 5 per group). Data in (**A**), (**B**) and (**D**) are presented as mean ± SEM. Each data point in (**B**) and (**D**) represents an individual mouse. Statistical significance was determined by two-way ANOVA followed by Sidak’s multiple-comparisons test (**A**) or two-tailed unpaired Student’s t-test (**B**, **D**).

To evaluate the effects of VPS4 inhibition on RMS growth, DN-VPS4A- or GFP-control-transduced KMR46 cells were intramuscularly injected into the left gastrocnemius of C57BL/6 mice. Beginning the day after inoculation, all mice were provided doxycycline-infused feed (625 ppm) to induce transgene expression. Tumor growth in the DN-VPS4A group was markedly reduced compared to the control GFP group (Fig. 7A). Consistently, tumors harvested at the experimental endpoint exhibited significantly reduced weight and volume in the DN-VPS4A group (Fig. 7B, C). These results are consistent with previous studies conducted under immunodeficient conditions and support the notion that targeting VPS4 autonomously induces cancer cell death, thereby suppressing RMS tumor growth [7, 18]. Notably, the tumor growth suppression induced by DN-VPS4A was accompanied by increased accumulation of various immune cells, including total T cells, CD4^+^ T cells, CD8^+^ T cells, total NK cells, cytotoxic NK cells, and NKT cells, along with a modest reduction in immunosuppressive myeloid-derived suppressor cells (MDSCs) (Fig. 7D). Monocyte and macrophage infiltration also trended higher in the DN-VPS4A tumors compared to the GFP tumors, although the increase did not reach statistical significance. These results suggest that under immunocompetent conditions, VPS4 inhibition may not only restrain RMS growth through autonomous cancer cell death, but also promote an immunogenic tumor microenvironment.

### Tumor-intrinsic STING facilitates the anti-tumor effects of VPS4 inhibition

**Fig. 8:**
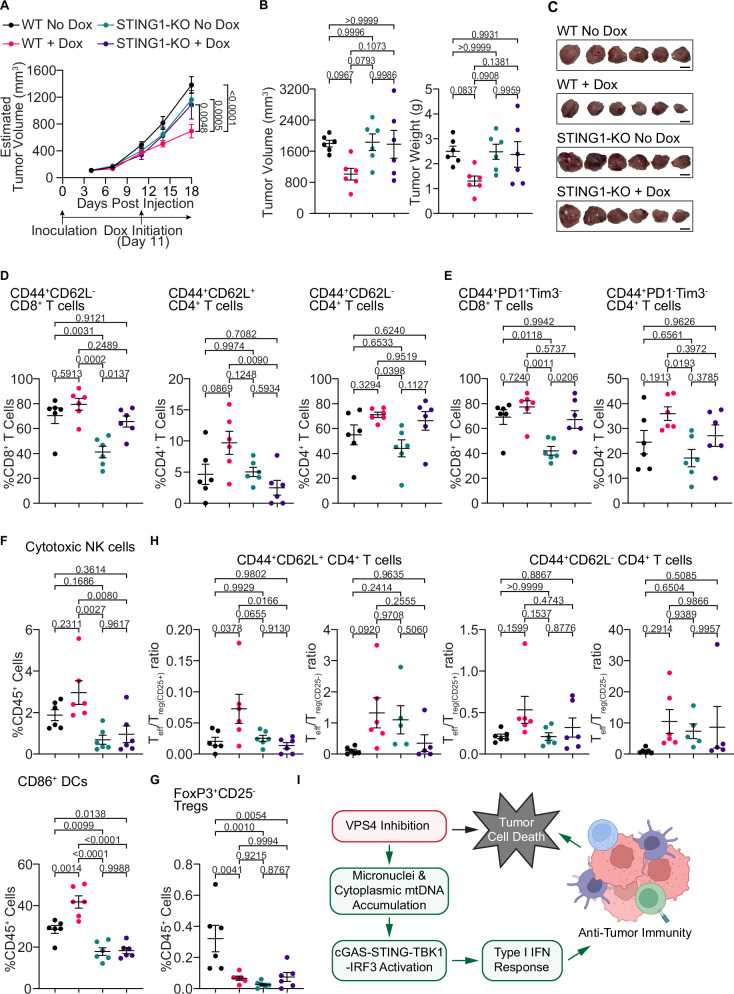
Tumor-intrinsic STING facilitates the anti-tumor effects of VPS4 inhibition and enhances antigen-presenting DC, NK cell, and effector T cell recruitment. **A** Tumor growth curves of doxycycline (Dox)-inducible DN-VPS4A-transduced KMR46 WT or STING1-knockout (KO) syngeneic allografts in immunocompetent C57BL/6 mice, treated with or without Dox-infused feed (625 ppm) starting on day 11 post injection (*n* = 6 per group). **B** Tumor volumes and weights at the experimental endpoint (1 week post Dox-treatment; 18 days post injection). **C** Representative images of harvested tumors. Scale bar represents 1 cm. **D** Flow cytometry quantification of tumor-infiltrating effector/effector memory (CD44^+^CD62L^-^) and central memory (CD44^+^CD62L^+^) T cell subsets (*n* = 6 per group). **E** Quantification of CD44^+^ effector T cells with or without exhaustion markers PD1 and Tim3 (*n* = 6 per group). **F** Quantification of cytotoxic NK cells and antigen-presenting (CD86^+^) dendritic cells (*n* = 6 per group). **G** Quantification of CD25^-^ regulatory T cells (*n* = 6 per group). **H** Ratios of effector CD4^+^ T cell subsets to CD25^+^ or CD25^-^ regulatory T cells within harvested tumors. **I** Schematic model of anti-tumor immunity induced upon VPS4 inhibition. Data in (**A**), (**B**), (**D–H**) are presented as mean ± SEM. Each data point in (**B**) and (**D–H**) represents an individual mouse. Statistical significance was determined by two-way ANOVA followed by Tukey’s multiple-comparisons test (**A**) or one-way ANOVA followed by Tukey’s multiple-comparisons test (**B**, **D–H**). In (**A**), (**B**), and (**D–H**), samples are color-coded as follows: wild-type (WT) without doxycycline (Dox) in black, WT + Dox in magenta, STING knockout (STING1-KO) without Dox in teal, and STING1-KO + Dox in purple.

If VPS4 inhibition enhances tumor immunogenicity via activation of the STING signaling axis, its anti-tumor effects should be diminished in the absence of STING. To test this, C57BL/6 mice were injected with DN-VPS4A-transduced KMR46 wild-type (WT) or STING1-KO cells. Once tumors reached 300-500 mm^3^, half of each group received doxycycline-infused feed, and tumors were harvested after one week. We observed that one week of doxycycline treatment reduced tumor growth in WT tumor-bearing mice, but had no significant effect on STING1-KO tumors (Fig. 8A). Ex vivo measurement of tumor volume and weight confirmed these findings (Fig. 8B, C). Time-lapse imaging of WT and STING1-KO in vitro cultures revealed comparable onset of cell death upon doxycycline treatment (Supplementary Fig. 1). This, together with the RNA-seq data (Fig. 5C) rules out the possibility that STING loss impairs DN-VPS4A-induced autonomous cell death. Together, these results indicate that tumor-intrinsic STING is a critical mediator of the anti-tumor effects induced by VPS4 inhibition in this RMS model.

Given the role of tumor cell-intrinsic STING signaling in enhancing antigenicity and promoting T cell activation, we performed flow cytometric analysis of tumor-infiltrating immune cells using expanded antibody panels. The myeloid panel included markers for dendritic cells (DCs) and CD86^+^ antigen-presenting cells, while the lymphoid panel assessed activation and memory markers (CD44, CD62L) alongside exhaustion markers (PD1, Tim3). A separate panel was used to quantify regulatory T cells within tumor cell suspensions. We found that effector/effector memory CD8^+^ T cells (CD44^+^CD62L^-^) were more abundant in WT than STING1-KO tumors, though doxycycline treatment increased their infiltration in both groups (Fig. 8E). Central memory CD4^+^ T cells (CD44^+^CD62L^+^), crucial for sustained anti-tumor immunity, were induced by doxycycline only in WT tumors (Fig. 8E). In contrast, effector memory CD4^+^ T cells (CD44^+^CD62L^-^) increased with doxycycline treatment regardless of STING1 status (Fig. 8E). Additionally, doxycycline enhanced the proportion of PD1^+^Tim3^-^ and PD1^-^Tim3^-^ subsets among activated CD44^+^ T cells, suggesting reduced T cell exhaustion upon VPS4 inhibition (Fig. 8F).

We also observed that cytotoxic NK cell infiltration increased in doxycycline-treated WT tumors but not in STING1-KO tumors (Fig. 8G), indicating STING-dependent recruitment. Because effector T cells, memory T cells, and NK cells depend on antigen-presenting cells for activation, we examined DC infiltration. Indeed, CD86^+^ DCs were enriched in doxycycline-treated WT tumors but not in STING1-KO tumors (Fig. 8G), suggesting that VPS4 inhibition promotes antigen presentation via STING-dependent mechanisms. To further explore effector T cell recruitment, we analyzed regulatory T (T_reg_) cells. Doxycycline-treated WT tumors exhibited reduced infiltration of CD25^-^ T_reg_ cells, a reservoir pool for CD25^+^ T_reg_ cells, compared to untreated WT tumors (Fig. 8H). Correspondingly, the ratios of central memory and effector/effector memory CD4^+^ T cells to T_reg_ cells increased in doxycycline-treated WT tumors but remained unchanged in STING1-KO tumors (Fig. 8I). Collectively, these findings demonstrate that VPS4 inhibition reshapes the tumor microenvironment towards a more immunogenic state, primarily through STING-dependent enhancement of antigen presentation and effector T cell recruitment. The corresponding tumor growth data further highlight STING as a key mediator of the anti-cancer efficacy achieved by targeting VPS4.

## Discussion

The ESCRT enzyme VPS4 has emerged as an attractive anti-cancer target, as cancer cells frequently lose one VPS4 paralog and become dependent on the remaining one for survival [21]. In this study, using a syngeneic mouse model of fusion-negative RMS [6, 7, 18], we demonstrate that targeting VPS4 not only induces cancer cell death, but also activates cytokine production and innate immune signaling, thereby enhancing anti-tumor immunity (Fig. 8I). Mechanistically, VPS4 inhibition drives transcriptional upregulation of inflammatory cytokines predominantly through the STING-TBK1-IRF3 axis and promotes immune cell infiltration into tumors. Conversely, loss of STING1 impairs these immune responses and diminishes the resulting anti-tumor effects.

Our findings also identify EtBr-sensitive mtDNA, sensed by cGAS, as a key trigger of cytokine upregulation following VPS4 inhibition. DN-VPS4 induction reduced mitochondrial membrane potential, suggesting that compromised mitochondrial membrane integrity underlies the increase in cytoplasmic mtDNA. Although the upstream mechanisms driving this process remains to be determined, it has been shown that ESCRT inhibition through depletion of the ESCRT-III subunit CHMP2A induces ER stress, leading to upregulation of BH3-only proteins including PMAIP1 [25], which in turn promote mitochondrial outer membrane permeabilization (MOMP) via BAX and BAK by antagonizing anti-apoptotic BCL-2 family proteins [41]. Consistently, we detected upregulation of ATF3, DDIT3, and PMAIP1 following DN-VPS4A induction. Given that BAX/BAK-mediated MOMP leads to mitochondrial inner membrane permeabilization and subsequent mtDNA efflux [42, 43], VPS4 inhibition may promote cytosolic mtDNA release by triggering mitochondrial herniation. In parallel, VPS4 inhibition can impair the clearance of damaged mitochondria by blocking autophagosome closure [36], which may further contribute to cytosolic mtDNA accumulation. Thus, it is plausible that MOMP induction, combined with defective autophagic clearance, leads to cytoplasmic exposure of mtDNA and subsequent activation of STING upon VPS4 inhibition.

In addition to STING signaling, our transcriptomic analysis revealed that VPS4 inhibition also upregulates NF-κB, stress response, and apoptosis pathways in a STING-IRF3-independent manner. Correspondingly, upregulation of certain immune signaling receptors, as well as induction of cell death, occurred even in STING1-deficient cells upon DN-VPS4A expression. These observations align with previous studies using STING-negative U-2 OS osteosarcoma [25, 44] and STING signaling-silenced HCT116 colorectal carcinoma [6, 44] cells, underscoring the diverse roles of ESCRT. Interestingly, in HCT116 cells, VPS4 depletion-induced NF-κB activation and cell death have been shown to drive macrophage activation and polarization towards the inflammatory M1 phenotype in vitro [6]. Moreover, although STING1-deficient RMS tumors exhibited reduced tumor suppression and impaired NK cell and DC infiltration following VPS4 inhibition, a subset of T cells still infiltrated in response to DN-VPS4A expression. Given that STING pathway silencing occurs in certain types of human cancers [13], it will be important to investigate how STING-independent immune mechanisms contribute to tumor progression upon VPS4 inhibition.

In summary, we demonstrate that targeting VPS4 induces anti-tumor immunity primarily through the STING pathway. While our findings reinforce the therapeutic potential of VPS4 as an anti-cancer target, no VPS4 inhibitors are currently available for clinical use. Interestingly, interferons, which are major downstream products of STING signaling, have been shown to downregulate VPS4 expression [7]. Furthermore, consistent with the role of the ESCRT machinery in mediating the termination of STING signaling [11, 12], we found that STING agonists further enhanced the cytokine upregulation induced by VPS4 inhibition. Together, these results suggest that combining interferons with STING agonists may represent a promising strategy to activate and enhance anti-tumor immunity, particularly in cancers with VPS4A or VPS4B deletion. Notably, despite a significant delay in tumor growth, RMS tumors were not completely abolished upon DN-VPS4 induction. Similar findings have been reported in other cancer types, where co-depletion of VPS4A and VPS4B also resulted in only partial growth suppression [6, 7]. These observations suggest that cancer cells may employ bypass mechanisms to tolerate VPS4 inhibition. Further studies are warranted to identify such mechanisms and maximize the therapeutic potential of targeting VPS4 in cancer.

## Supplementary information

SUPPLEMENTARY MATERIAL

SUPPLEMENTARY TABLES

## Data Availability

RNA-sequencing data generated in this study have been deposited in the NCBI Gene Expression Omnibus (GEO) under accession number GSE328591. All other data are available upon request.
