# Supplementary material for: Targeting VPS4 elicits STING-driven anti-tumor immunity to suppress rhabdomyosarcoma growth
Source: Oncogene. 2026 Apr 24;45(23):2211–24. doi: 10.1038/s41388-026-03800-1 (PMC13246437; doi:10.1038/s41388-026-03800-1)
Supplement: Supplementary file 1 — SUPPLEMENTARY MATERIAL [file 41388_2026_3800_MOESM1_ESM.docx]

**SUPPLEMENTARY MATERIAL**

**Targeting VPS4 elicits STING-driven anti-tumor immunity to suppress rhabdomyosarcoma growth**

Ray Zhang^1^, Longgui Chen^1^, Xinwen Liang^1^, Jiawen Zhang^1^, Kouta Hamamoto^1^, Tatsuya Hattori^1,2^, Venugopal Vangala^1^, Todd Schell^2^, Giselle Saulnier Sholler^1^, Yoshinori Takahashi^1,2^, Hong-Gang Wang^1,2^

^1^Division of Pediatric Hematology and Oncology, Department of Pediatrics, ^2^Department of Cell and Biological Systems, The Pennsylvania State University College of Medicine, Hershey, Pennsylvania, USA

**Supplementary Methods**

**Generation of cell lines with STING1 or IRF3 knockouts**

KMR19 and KMR46 DN-VPS4 cells were first transduced with lentiviral particles harboring Cas9 (Addgene #52962) followed by blasticidin selection (10 µg/mL). The Dharmacon Edit-R system from Horizon Discovery Ltd. was subsequently used per manufacturer’s instructions to transfect the Cas9-expressing RMS cell derivatives with sgRNAs targeting STING1 or IRF3. To generate STING1 knockouts, three pre-made sgRNAs targeting STING1 from Horizon Discovery Ltd. (SG-055528-01-0010, SG-055528-02-0010, SG-055528-03-0010) were administered into separate cell cultures. To generate IRF3 knockouts, three pre-made sgRNAs targeting IRF3 from Horizon Discovery Ltd. (SG-041095-01-0010, SG-041095-02-0010, SG-041095-03-0010) were administered into separate cell cultures. Knockout of STING1 or IRF3 was verified by immunoblotting cell lysates with anti-STING antibody (Cell Signaling Technology #13647S, 1:1000) or anti-IRF3 antibody (Cell Signaling Technology #4302S, 1:1000).

**Generation of KMR46 VPS4B knockout cell line**

To generate KMR46 VPS4B-knockout cells, oligos targeting VPS4B were first ligated into empty epiCRISPR plasmid (Addgene #135960) at the SapI cutsite. Four VPS4B-targeting complementary oligo pairs were designed: #1 (5’-CCGTAAAGCCAAGCAAAGTATCA-3’; 5’-AACTGATACTTTGCTTGGCTTTA-3’), #2 (5’-CCGCATACCCCTTCTCATCAACT-3’; 5’- AACAGTTGATGAGAAGGGGTATG-3’), #3 (5’-CCGGGCTGCACGGAGAATTAAGA-3’; 5’-AACTCTTAATTCTCCGTGCAGCC-3’), and #4 (5’-CCGGGAAAGCGGACACCTTGGAG-3’; 5’-AACCTCCAAGGTGTCCGCTTTCC-3’). Successful insertion of the VPS4B-targeting oligos into the epiCRISPR plasmid was confirmed by sequencing. Afterwards, KMR46 cells were transfected with the VPS4B-epiCRISPR plasmids using Lipofectamine 3000 Transfection Reagent (Thermo Fisher Scientific #L3000001) as per manufacturer’s instructions followed by puromycin selection (10 µg/mL). The selected, transfected KMR46 derivative cells underwent single cell cloning, and VPS4B-knockout clones were verified by immunoblotting of cell lysates with anti-VPS4B antibody (Santa Cruz Biotechnology #sc-377162, 1:200).

**Construction of KMR46 VPS4B knockout cells with inducible depletion of VPS4A by shRNAs**

KMR46 VPS4B knockout cells were transduced with lentivirus particles harboring doxycycline-inducible shRNA targeting VPS4A (shVPS4A) followed by blasticidin selection (10 µg/mL).

To make inducible shVPS4A expression plasmid, the puromycin-resistance gene in the shRNA Cloning and Expression Vector from Cellecta (#SVSHU6T1G-L; pRSITEP-U6Tet-sh-EF1-TetRep-2A-Puro) was first replaced with a blasticidin-resistance gene (P2A-Blast). Successful generation of the pRSITEP-U6Tet-sh-EF1-TetRep-P2A-Blast vector was validated by sequencing. Afterwards, the VPS4A-targeting complementary oligo pairs VPS4A #1 (5’-accgGGCAAGAAGCCAGTCAAAGAGAGTTAATATTCATAGCTCTCTTTGACTGGCTTCTTGCTTTT-3’; 5’-cgaaAAAAGCAAGAAGCCAGTCAAAGAGAGCTATGAATATTAACTCTCTTTGACTGGCTTCTTGCC-3’), VPS4A #2 (5’-accgGCGAGAAGCTGAAGGATTATTTGTTAATATTCATAGCAAATAATCCTTCAGCTTCTCGTTTT-3’; 5’-cgaaAAAACGAGAAGCTGAAGGATTATTTGCTATGAATATTAACAAATAATCCTTCAGCTTCTCGC-3’) and VPS4A #3 (5’-accgGGCCGAGAAGCTGAAGGATTATGTTAATATTCATAGCATAATCCTTCAGCTTCTCGGCTTTT-3’; 5’-cgaaAAAAGCCGAGAAGCTGAAGGATTATGCTATGAATATTAACATAATCCTTCAGCTTCTCGGCC-3’) were designed. The VPS4A #1, #2, and #3 oligo pairs were subsequently ligated into the pRSITEP-U6Tet-sh-EF1-TetRep-P2A-Blast vector as per manufacturer’s instructions for the normal Cellecta shRNA Cloning and Expression Vector, in turn generating the inducible shVPS4A expression plasmids shVPS4A #1, #2, and #3 respectively. All shVPS4A plasmids were confirmed using sequencing.

**Confocal Microscopy**

Cells were grown on Lab-Tek II Chambered Coverglass (Fisher Scientific #155409), fixed in 4% paraformaldehyde for 10 min, permeabilized with 0.2% Triton X-100 for 10 min, blocked in 10% normal goat serum for 1 h, and then incubated with phospho-STING (Ser365) primary antibody (Thermo Fisher Scientific #62912, 1:50) followed by Alexa Fluor 647-conjugated goat anti-rabbit secondary antibody (Invitrogen #A21244). Nuclei were counterstained with DAPI (BD Biosciences [Franklin Lakes, NJ, USA] #564907). Fluorescent images were obtained using a Leica AOBS SP8 laser-scanning confocal microscope (63× oil-immersion lens), deconvolved using Huygens deconvolution software (Scientific Volume Imaging), and analyzed using Imaris software (Bitplane) or Volocity software (PerkinElmer).

**Micronuclei quantification by flow cytometry**

The protocol for micronuclei staining and gating strategy was described previously(1,2). Briefly, 5 x 10^5^ to 1 x 10^6^ cells were pelleted and resuspended in 300 µL of 10 µg/mL ethidium monoazide (Thermo Fisher Scientific #E1374) diluted in PBS plus 2% FBS. Cells were incubated on ice for 30 min under cool white light for photoactivation, then pelleted and washed once with PBS plus 2% FBS. Cells were resuspended in Lysis Buffer 1 (1 mg/mL Trisodium citrate; 0.584 mg/mL NaCl; 0.6 µL/mL IGEPAL; 100 µg/mL RNase A; 0.5 µM SYTOX Red [Thermo Fisher Scientific #S34859]; prepared in MilliQ water and filtered through 0.22 µm filter) and incubated in the dark for 1 h at RT. Without removing Lysis Buffer 1, 300 µL of Lysis Buffer 2 (10 mg/mL citric acid; 85.6 mg/mL sucrose; 0.5 µM SYTOX Red; prepared in MilliQ water and filtered through 0.22 µm filter) was added. Samples were incubated in the dark for an additional 30 min at RT, then stored in 4°C until analyzed via flow cytometry.

**Cytosolic mtDNA Quantification**

The cytosolic fraction of KMR46 cells was isolated as described previously(3) and used for cytosolic DNA extraction with the ChIP DNA Clean & Concentrator kit (Zymo Research [Tustin, CA, USA] #D5205), following the manufacturer’s instructions. Total cell DNA was extracted from whole cell lysate (WCL) fractions using the QIAamp DNA Mini Kit (Qiagen #51304) according to the manufacturer’s instructions. Target genes were amplified using a standard qPCR protocol: template DNA was diluted 1:8 and mixed with iTaq Universal SYBR Green Supermix (Bio-Rad Laboratories [Hercules, CA, USA], #1725121), 10 µM forward and reverse primers, and sterile distilled water. Primers for D-loop (Forward: AATCTACCATCCTCCGTGAAACC; Reverse: TCAGTTTAGCTACCCCCAAGTTTAA), Cox1 (Forward: GCCCCAGATATAGCATTCCC; Reverse: GTTCATCCTGTTCCTGCTCC), and Cytochrome B (Forward: GCTTTCCACTTCATCTTACCATTTA; Reverse: TGTTGGGTTGTTTGATCCTG) (4,5) were synthesized by Eurofins Genomics (Louisville, KY, USA). GAPDH (Forward: CGTTGAATTTGCCGTGAGTG; Reverse: CACTACAGACCCATGAGGAGT) was amplified to measure potential nuclear DNA contamination in the cytosolic fraction. Amplification and data collection were performed on a Bio-Rad CFX96 Real-Time System using the following program: 95°C for 2 min (initial denaturation), followed by 40 cycles of 95°C for 5 s and 60°C for 30 s, and a final melt curve step. All samples were run in triplicate, and assays were repeated at least three times. Quantification cycle (Cq) values for cytosolic mtDNA genes were normalized to the respective Cq values of WCL mtDNA genes, and data were expressed as fold change over vehicle-control sample.

**Mitochondria Membrane Potential**

Assessment of mitochondrial membrane potential and depolarization was conducted using the MitoProbe TMRM Assay Kit for Flow Cytometry (Thermo Fischer Scientific #M20036). KMR46 cells were incubated in 200 nM TMRM diluted in serum-free RPMI for 1 h before being stained with Annexin V (APC, Biolegend #640941) and analyzed via flow cytometry.

**Depleting cGAS using siRNAs**

To deplete cGAS from KMR46 DN-VPS4 cells, custom siRNAs targeting cGAS were first designed and synthesized by Horizon Discovery Ltd. The oligonucleotide sequences of si-cGAS #1 (5’-GGAUUGAGCUACAAGAAUA-3’) and si-cGAS #2 (5’-GCUGUAACACUUCUUAUCA-3’) were described previously(6), and were synthesized with a dTdT overhang on the 3’-end. KMR46 cells were then transfected with 10 µM of si-cGAS #1, si-cGAS #2, or non-specific scramble siRNA using Lipofectamine RNAiMAX transfection reagent (Thermo Fisher Scientific #13778150) and protocols provided by the manufacturer. Depletion of cGAS was assessed by immunoblotting of cell lysates with anti-cGAS antibody (Cell Signaling Technology #31659S, 1:1000).

**Antibody panels for tumor immune profiling by flow cytometry**

Lymphoid cell panel: CD3 (PE, BioLegend #100206), CD4 (AF700, BioLegend #100536), CD8a (PE-Cy7, BioLegend #100722), CD45.2 (APC, BioLegend #103112), NK1.1 (BV421, BioLegend #108732). For the expanded lymphoid panel, the following were also added: CD44 (PerCP-Cy5.5, BioLegend #103032), Tim3 (BV711, BioLegend #134021), PD1 (BV605, BioLegend #135220), CD62L (BV650, BioLegend #104453). Following antibody staining, cells were washed twice with FACS buffer and fixed with 2% paraformaldehyde at RT for 10 min in the dark.

Myeloid cell panel: CD45.2 (BV421, BioLegend #103134), CD11b (BV711, BioLegend #101241), F4/80 (BV605, BioLegend #123133), Ly6G (AF700, BioLegend #127622), Ly6C (PE, eBioscience #12-5932-82). For the expanded myeloid panel, the following were also added: CD86 (BV650, BioLegend #105036), CD11c (APC, BioLegend #117309), MHCII I-A/I-E (PerCP, BioLegend #107624).

Regulatory T cell panel: Surface staining was performed using antibodies against CD45.2 (BV421, BioLegend #103134), CD4 (AF700, BioLegend #100536), CD3 (PE, BioLegend #100206), and CD25 (BV711, BioLegend #102049). Intracellular staining for FoxP3 (AF647, BioLegend #126408) was conducted according to manufacturer’s instructions using the FoxP3/Transcription factor staining buffer kit (Invitrogen #00-5533).

**
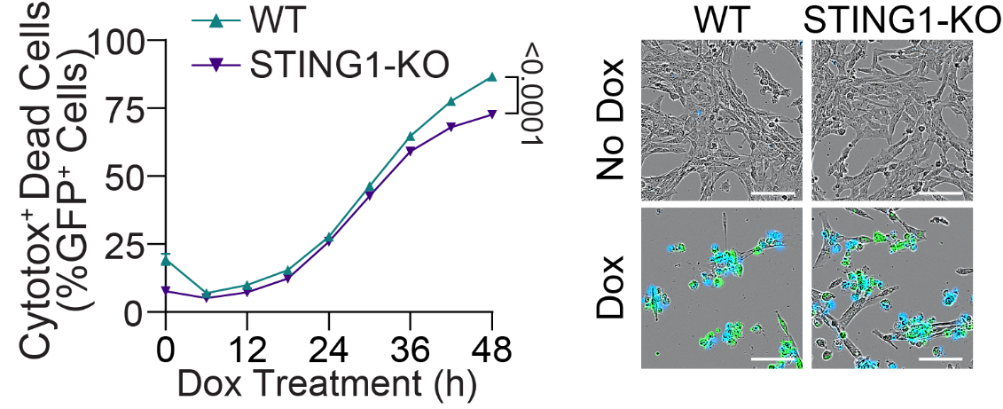
**

**Supplementary Figure 1. STING is dispensable for VPS4 inhibition-induced cell death.** Time lapse monitoring of cell death, marked by Cytotox positivity (blue), following induction of DN-VPS4A expression (GFP^+^; green) by Dox in KMR46 WT and STING1-KO cells (left). Representative images at 48 h are shown (right). Scale bar represents 100 µm.

**Table S1. Raw gene counts**

**Table S2. Limma-voom–normalized gene expression values**

**References**

1. Bryce SM, Bemis JC, Avlasevich SL, Dertinger SD. In vitro micronucleus assay scored by flow cytometry provides a comprehensive evaluation of cytogenetic damage and cytotoxicity. Mutat Res. 2007 Jun 15;630(1–2):78–91.

2. Vallabani NVS, Karlsson HL. Primary and Secondary Genotoxicity of Nanoparticles: Establishing a Co-Culture Protocol for Assessing Micronucleus Using Flow Cytometry. Front Toxicol. 2022;4:845987.

3. Bryant JD, Lei Y, VanPortfliet JJ, Winters AD, West AP. Assessing Mitochondrial DNA Release into the Cytosol and Subsequent Activation of Innate Immune-related Pathways in Mammalian Cells. Curr Protoc. 2022 Feb;2(2):e372.

4. Moriyama M, Koshiba T, Ichinohe T. Influenza A virus M2 protein triggers mitochondrial DNA-mediated antiviral immune responses. Nat Commun. 2019 Oct 11;10(1):4624.

5. Jahun AS, Sorgeloos F, Chaudhry Y, Arthur SE, Hosmillo M, Georgana I, et al. Leaked genomic and mitochondrial DNA contribute to the host response to noroviruses in a STING-dependent manner. Cell Rep. 2023 Mar 28;42(3):112179.

6. Mackenzie KJ, Carroll P, Lettice L, Tarnauskaitė Ž, Reddy K, Dix F, et al. Ribonuclease H2 mutations induce a cGAS/STING-dependent innate immune response. EMBO J. 2016 Apr 15;35(8):831–44.
